# Pathogenetic Aspects of Systemic Sclerosis: A View Through the Prism of B Cells

**DOI:** 10.3389/fimmu.2022.925741

**Published:** 2022-06-23

**Authors:** Konstantinos Melissaropoulos, George Iliopoulos, Lazaros I. Sakkas, Dimitrios Daoussis

**Affiliations:** ^1^ Department of Rheumatology, Agios Andreas Hospital, Patras, Greece; ^2^ Department of Rheumatology , University of Patras Medical School, Patras University Hospital, Patras, Greece; ^3^ Department of Rheumatology and Clinical Immunology, Faculty of Medicine, School of Health Sciences, University of Thessaly, Larissa, Greece

**Keywords:** B cells, systemic sclerosis, scleroderma, fibrosis, immune system

## Abstract

Systemic sclerosis (SSc) is a rare fibrotic rheumatic disease, associated with psychological distress and increased morbidity and mortality due to skin involvement and internal organ damage. The current understanding of the complex pathogenesis is yet incomplete and disease therapeutic algorithms are far from optimal. Immunologic aberrations are considered key factors for the disease, along with vascular involvement and excess fibrosis. Adaptive immunity and its specialized responses are an attractive research target and both T and B cells have been extensively studied in recent years. In the present review, the focus is placed on B cells in SSc. B cell homeostasis is deranged and B cell subsets exhibit an activated phenotype and abnormal receptor signaling. Autoantibodies are a hallmark of the disease and the current perception of their diagnostic and pathogenetic role is analyzed. In addition, B cell cytokine release and its effect on immunity and fibrosis are examined, together with B cell tissue infiltration of the skin and lung. These data support the concept of targeting B cells as part of the therapeutic plan for SSc through well designed clinical trials.

## Introduction

Systemic sclerosis (SSc) is a rare rheumatic disease manifesting with fibrosis of the skin and internal organs (1). The disease carries a significant burden for affected patients as exemplified by the major psychologic distress associated with functionality loss and appearance change along with the worse standardized mortality ratio among rheumatic diseases (2). Genetically predisposed individuals are exposed to environmental factors and various triggers to initiate a homeostasis dysregulation. Progression of the disease is characterized by pathophysiologic changes of the immune system and vasculature, which precede the fibrotic phenotype and emphasize the complex multisystem nature of the disease (3). Endothelial apoptosis, platelet activation, aberrant homeostasis of vasoactive molecules favoring vasoconstriction, underlie the vasculopathy of SSc, which is characterized by fibroproliferation of the vessel walls and capillary rarefaction (4). Macrophages, dendritic cells and innate lymphoid cells are cell components of innate immunity with pathogenetic implications for the disease. Moreover, T and B cell disturbed homeostasis is believed to be critical for SSc pathogenesis and evolution (5). Genome wide association studies have identified associated genetic variants in recent years, highlighting the role of the immune system. Regarding B cells, variants of the molecules B lymphocyte kinase (BLK) and B cell scaffold protein with ankyrin repeats 1 (BANK1) have been linked to SSc (6). In addition, B cells are overall activated in patients with the disease and aberrations of cell signaling pathways have been described. Autoantibodies produced by B cells characterize connective tissue diseases. The presence of SSc specific autoantibodies facilitates diagnosis and dictates prognosis of SSc but their role in the pathogenesis of the disease is still a subject of ongoing research (7). Endothelial cell damage and direct fibroblast activation are among the speculated autoantibody mediated effects linking the pathophysiologic triplet of the disease (autoimmunity-vasculopathy-fibrosis). In addition, activated B cells infiltrate involved organs, interact locally with immune and mesenchymal cells and can be a source of major proinflammatory and profibrotic cytokines such as IL-6 and TGF-β. These data highlight the multipotential facets of B cell function in SSc and justifies the clinical research of B cell targeting agents, such as the B cell depleting drug rituximab, in patients with the disease.

## Methods

We performed an electronic search in PubMed from inception until February 2022 using the following key words: systemic sclerosis, scleroderma and B cell in all combinations. We included articles only in English language and even though no time limit was set we focused on articles published during the last 15 years. An additional manual search was performed on the reference lists of retrieved articles. We focused our search on articles related to the potential role of B cells in fibrosis in both experimental models of scleroderma and patients with SSc.

## Results

### B Cell Aberrant Homeostasis in SSc

#### Phenotypical Changes in Circulating B Cells in SSc

Evidence indicates that B cells exhibit an aberrant phenotype in the peripheral blood of patients with SSc. The memory B cell compartment appears to be diminished though activated and the naïve population is expanded, potentially in a compensatory manner (8). In addition, the cytokine system that controls the survival of B cells, consisting mainly of the B cell activating factor (BAFF) and A Proliferation-Inducing Ligand (APRIL), is upregulated in the serum of patients with SSc (9, 10). Since these early studies, more research teams have also studied SSc patients, confirming the basic notion of B cell activation and trying to apply analytical immunophenotyping. Memory B cells indeed express activation and pro-apoptotic surface markers, such as CD86, CD95 and HLA-DR (11–13). Among the memory B cell subset, researchers have identified expanded effector cell populations, such as the double negative CD27-IgD- B cells (14) and the CD19+IgD−CD27+CD38−CD95+ activated switched memory B cells (15). The latter population was found to be increased in patients with diffuse cutaneous SSc (dcSSc) and patients with interstitial lung disease (ILD). Another B cell subset of special interest in autoimmune and infectious diseases are the CD21^low^ B cells, which exhibit high levels of activation markers, increased antigen presenting potential and belong primarily to the memory B cell compartment (16). In SSc, this population has been found expanded, perhaps indicating a subset with increased effector potential and enriched in autoreactive B cell clones (12, 17, 18). From a clinical point of view, increased CD21^low^ B cell numbers correlated with vascular complications in SSc, such as pulmonary arterial hypertension and new digital ulcers (17, 19).

B regulatory (Bregs) cells are a distinct IL-10 producing B cell subtype enriched in CD24^high^CD38^high^ transitional and CD24^high^CD27+ memory B cells. This cell population is believed to exert a suppressive effect on inflammation and autoimmunity (20). In patients with SSc, circulating Bregs were found decreased and functionally impaired after TLR-9 stimulation (21). It is interesting that their levels were negatively correlated with anti-topo I and anti-centromere autoantibody titers, circulating T follicular helper cell numbers and the overall disease activity (22, 23).

The diversity and rarity of the disease represents a challenge regarding the immunophenotyping of these patients. New technological advances, such as mass cytometry, make possible the simultaneous detection of a large number of markers and are now used to identify clusters of patients corresponding to specific cell signatures (24, 25). Different stages of fibrotic disease (early vs late), clinical phenotypes (diffuse vs limited disease) and the presence of distinct clinical characteristics such as the presence of ILD, are separately examined to reveal differences by the means of these new methods. New technologies can give insight into the interaction of B cells with other cells of the immunome in specific subgroups of patients. For the time being, overactivation is recorded in the peripheral blood of patients with SSc arising from an imbalance of effector and regulatory cell phenotypes.

#### B Cell Signaling in SSc

**Figure 1:**
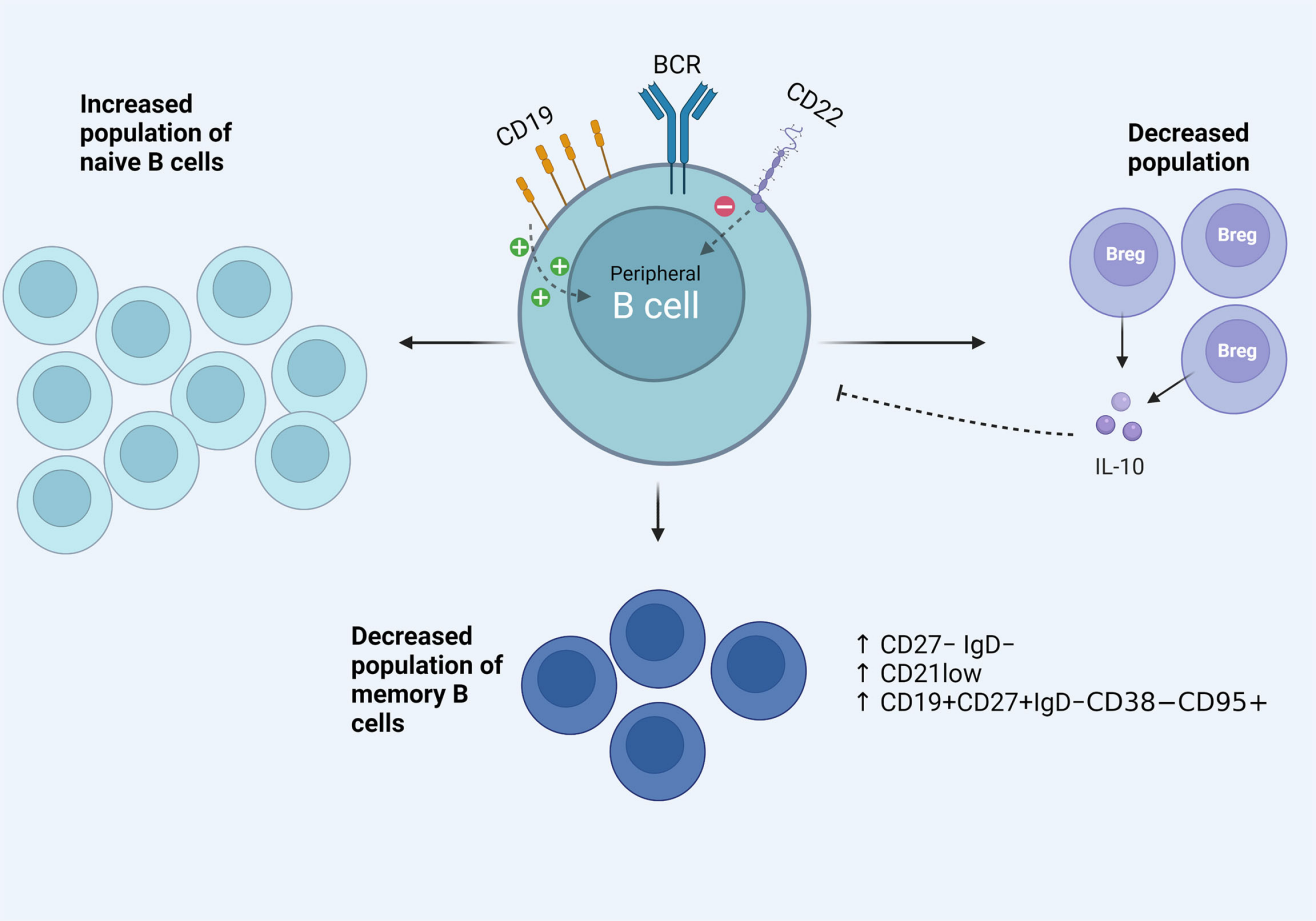
Aspects of the activated B cell phenotype in the peripheral blood of patients with SSc. Signaling defects including CD19/CD22 imbalance, increased effector B cell phenotypes among the memory B cell compartment and decreased B regulatory population and function.

B cell receptor (BCR) is a membrane-bound immunoglobulin on the cell surface and serves as the main receptor in B cell biology. BCR is necessary for the survival and development of early B cells, as well as antigen recognition in the periphery. Various co-receptors fine tune its function and multiple downstream pathways mediate the final effect on the cell nucleus and gene expression (26, 27). Activated B cell status in SSc could be partly explained by specific signaling defects. CD19 and CD22 are membrane glycoproteins and exert a positive and negative role in BCR signaling, respectively (28). CD19 was found to be upregulated in B cells of patients with SSc (11, 21, 29), with approximately 20% overexpression in the cohort studied by the first research team reporting it (29). In addition, our research group has found CD22 underexpression in a cohort of patients with diffuse cutaneous SSc, especially those with ILD. Subsequent experiments showed that CD22 phophorylation upon BCR stimulation was also defective in these patients (30). It seems that the imbalance of the CD19/CD22 system is part of the activated B cell phenotype in SSc. FcγRIIb is an inhibitory receptor of B cells, increasing the activation threshold of BCR. In a recent study, the levels of FcγRIIb were studied in B cell subsets of patients with SSc and were upregulated in the naïve and double negative B cell compartment (13). This could indicate homeostatic changes in signaling regulators of B cells and highlights the need for analytical immunophenotyping and signaling research in targeted effector B cell subsets in the future. Therapeutic targeting of key signaling molecules of B cells, such as the Bruton’s tyrosine kinase (BTK), is already employed in hematology (31) and research efforts show promising results in samples from patients with SSc. Einhaus et al. isolated B cells from PBMCs of 24 patients with SSc and performed *in vitro* experiments (32). They found that treatment with ibrutinib, a BTK inhibitor, resulted in attenuation of profibrotic cytokine IL-6 in B cell culture supernatants and also inhibited the toll like receptor mediated downstream activation of the transcription factor NFκB. The above data indicate that B cell signaling pathways in SSc are deranged. Further research could identify target molecules with therapeutic potential. Phenotypical changes and signaling aberrations in SSc B cells are diagrammatically depicted in **Figure 1**.

#### B Cell Infiltration in Affected Tissues

Lung involvement is a dreaded complication of SSc. Lafyatis et al. studied lung tissue of 11 patients with SSc-associated ILD. B cell infiltration, often in lymphoid aggregates, was shown with immunohistochemical staining (33). A small number of lung samples by patients with SSc and pulmonary arterial hypertension also confirmed B cell infiltration (34). Moreover, in another study aiming to associate bronchoalveolar lavage fluid (BALF) characteristics and ILD progression, the authors reported worse outcome in patients with a higher CD19+ percentage count in BALF (35).

Skin involvement in the disease characterizes the clinical phenotype of these patients. Histologic evaluation of the skin is relatively simple and studies have long identified immune cell infiltrates in affected skin, consisting of plasma cells, lymphocytes and macrophages (36, 37). Lymphocyte infiltrates were mostly attributed to T cells (37). However, it was Whitfield et al. who identified a strong B cell signature in affected and unaffected skin of a small cohort of four diffuse SSc patients compared with matched healthy individuals. Researchers used gene expression analysis employing DNA microarrays technology, as well as immunohistochemistry to manifest CD20+ B cell infiltrates in SSc skin. Immunoglobulins and CD53 genes associated with B lymphocytes showed prominent expression in the samples studied (38). A subsequent study with a larger sample of patients, used the same technology and tried to identify gene expression patterns. B cells were found in few numbers along with B cell gene expression mostly in the inflammatory group, which is characterized by increased immune response and lymphocyte infiltration indices; however, T cells predominated in this specific cohort (39). It seems that T and B cell interplay is crucial in the immunopathogenesis of the disease. In another study, immunohistochemical analysis of scleroderma skin confirmed mononuclear cell infiltration consisting of T cells, macrophages, and B cells. Interestingly, B cell infiltration was correlated with worse skin progression. In addition, B cell numbers were greater in patients with early disease, implying that skin infiltration with B cells could be critical in the early stages of SSc (40). The latter is supported by the finding of upregulated BAFF mRNA expression in the skin of patients with early but not late disease (9). RNA sequencing and transcriptome analysis is implemented in recent studies. Skaug et al. studied 48 patients with early diffuse SSc and found more skin samples expressing genes indicative of immune cell infiltration compared with healthy controls and patients with longer disease duration. Besides T cell and macrophage signatures, B cell signature was evident in 69% of the patients studied (41). Single cell RNA sequencing has been recently applied in active scleroderma skin, focusing on the tissue resident and recirculating T cells, to better characterize T cell clusters in patients with the disease. Researchers identified a unique CD4+CXCL13+ T cell phenotype with T follicular helper-like gene expression. They used confocal immunofluorescence microscopy to visualize spatial localizations of T cell subsets, and it is interesting that this unique phenotype co-localized with CD20+ B cells within inflammatory infiltrates of the scleroderma skin. This implies that B and T cell interaction at the level of inflamed tissue drives B cell responses locally in a manner similar of ectopic lymphoid structure (42).

**Figure 2:**
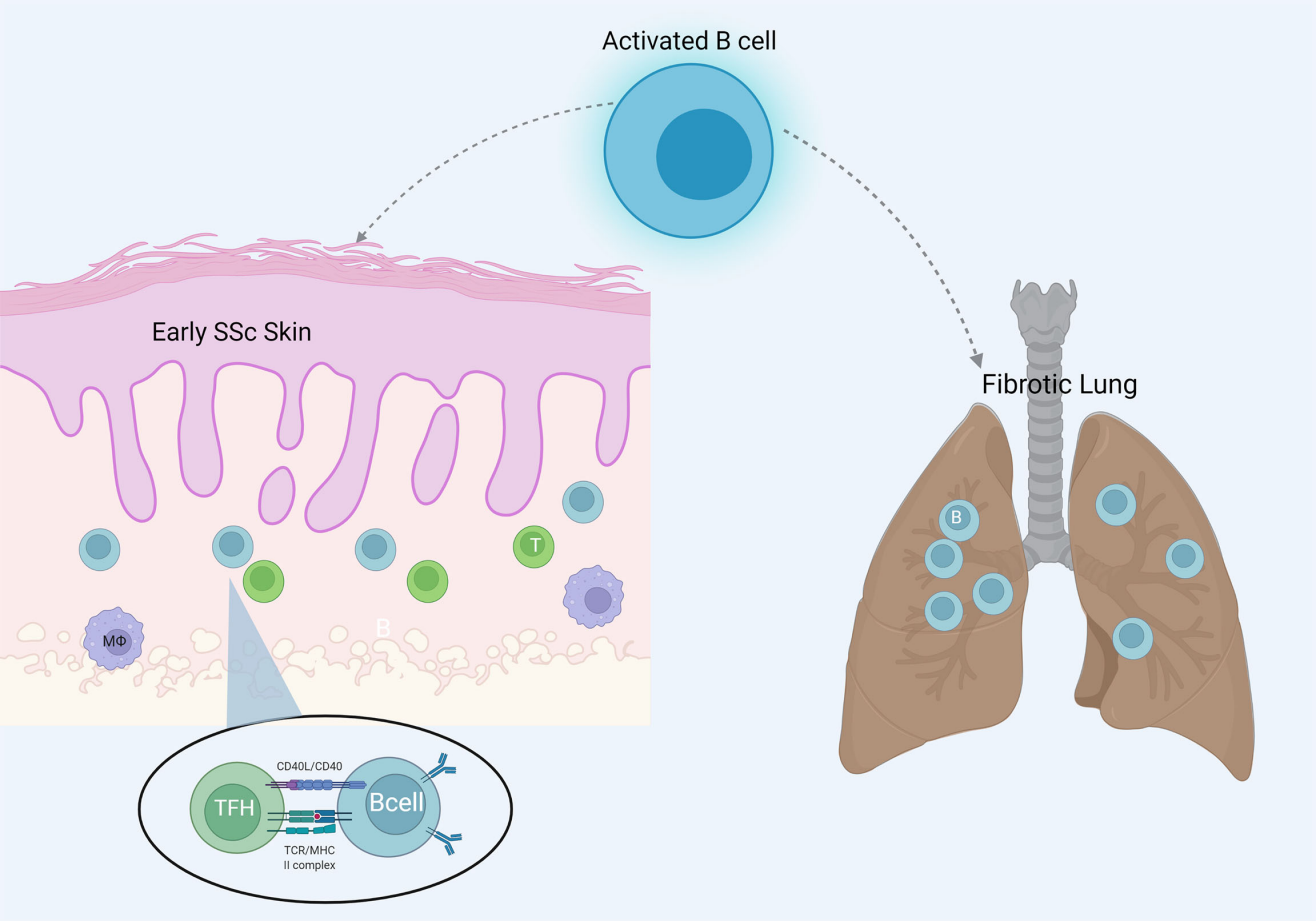
B cell infiltration of the target organs skin and lung in SSc. B cells are more evident in early skin lesions and interact locally with T cells resembling ectopic lymphoid structures.

The concept of studying the characteristics of the immune cells infiltrating target organ tissue, such as the lung and the skin, offers an important perspective of the disease. Ongoing evolution of the “omic” technologies even at a single cell level, can identify new immune phenotypes possibly critical for the pathophysiology of the disease. Overall, evidence points to the direction that target organs of the disease are infiltrated by immune cells, including B cells, especially at earlier disease stages, a finding with potential pathogenetic implications. B cell infiltration of affected tissues in SSc is shown in **Figure 2**.

### Pathogenetic Aspects of B Cells in SSc

#### SSc Specific Autoantibodies: From Diagnosis to Pathogenetic Implications

One of the most well-known functions of B cells is the production of antibodies. SSc is characterized by the presence of specific autoantibodies which have been extensively studied and used in everyday clinical practice for both diagnostic and prognostic purposes (43). There are 3 main SSc specific auto-Abs: anti-topoisomerase I antibodies (ATA), anti-centromere antibodies (ACA) and anti-RNA polymerase III antibodies (ARA). Several studies have shown many clinical and genetic associations of those auto-Abs to disease subtypes, organ related damage and even cancer (44)Recent genetic studies have revealed that SSc patients with different auto-Abs may have striking differences in skin gene expression, potentially indicating diverse pathophysiologic mechanisms (45). Similarly, another study investigated how gene expression profile in SSc skin relates to the presence of specific auto-Abs (46). Increased gene expression responsible for keratinocyte differentiation was found in ACA, cellular response to TGFβ and NFκβ signaling in ARA and response to cellular stress in ATA. These data point to the direction that auto-Abs may be used to differentiate patients in specific subsets with similar pathophysiology and therefore pinpoint patients more likely to respond to certain treatments. However, despite the fact that auto-Abs are strongly associated with clinical manifestations and skin gene expression, evidence indicating a direct pathogenetic role is limited.

ACAs target centromere proteins (CENPs), which are nuclear proteins that take part in the formation of the kinetochore during cell division. Healthy subjects or patients with tumors that overexpress CENP-A are not positive for Anti–CENP-A antibodies (47), implying that the production of those antibodies is SSc specific. Recently, Kajio et al. explored the autoantigenic nature of centromere proteins in patients with SSc (48). They reported that ACAs target the protein complex of the centromere and kinetochore, but there was no proven or proposed mechanism as to how these auto-Abs could participate in SSc pathogenesis.

ATAs bind to topoisomerase I (Topo I), which is an enzyme that takes part in the ligation of DNA. Data indicate that ATAs can bind directly to the surface of fibroblasts in SSc patients, but it remains unknown whether this binding could lead to fibroblast activation (49). Another study associated the presence of IgM ATAs with disease progression in ATA (+) SSc patients, but no pathogenetic involvement has been yet established (50). Hénault et al. investigated how the binding of Topo I and ATAs to fibroblasts affected the behavior of monocytes when co-cultured (51). Through a series of experiments, the authors showed that Topo I could bind directly to fibroblasts and recruit ATAs. ATAs had the ability to bind to the aforementioned complex and this binding led to increased monocyte adhesion and activation. Furthermore, another research team isolated fibroblasts from both healthy and affected SSc skin (52). All fibroblasts were then stimulated with IgG ACAs and ATAs. The stimulation of SSc fibroblasts with IgG ACAs and ATAs led to a significant increase in profibrotic markers. These data may support the possible role of ATAs in disease pathogenesis.

Shen et al. incubated calf pulmonary arterial endothelial cells (CPAEs) with sera containing ACAs and ATAs from SSc patients (53). They found that ACAs and ATAs could accelerate endothelial cell aging, implying a potential involvement of those auto-Abs in SSc vasculopathy.

There are some hypotheses regarding potential pathogenetic mechanisms for both ATAs and ACAs. It is of interest that apoptotic endothelial cells may release CENP-B and topoisomerase I (TOPO I). CENP-B may bind to pulmonary artery smooth muscle cells (PASMCs) *via* CCR3. ACAs may bind to this complex and this could lead to vascular damage. On the other hand, dendritic cells (DCs) may carry Topo I, released from apoptotic endothelial cells and present it to T cells. When activated, T cells produce IL-2, IL-6 and communicate with B cells which in turn produce ATAs. ATAs may bind to fibroblasts possibly *via* CCR7 and the formed complex may recruit and activate monocytes, exacerbating fibrosis (54).

ARAs generally show different phenotypical and pathogenetic characteristics from ACAs and ATAs. The major concern is that SSc patients with ARA have a high risk of developing cancer (55). Polymerase III polypeptide A (POLR3A) gene is responsible for coding the RPC1 subunit of RNA polymerase. Joseph et al. reported that mutations of this gene were found in ARA(+) SSc patients with cancer and that these mutations were also responsible for T-cell and humoral immune responses. The authors propose that the immune response against a mutant tumor peptide could cross react with the normal form of this protein and contribute to the pathogenesis of the autoimmune disease (56). These data highlight the involvement of cellular immunity, and the findings overall might imply a possible role of ARAs in SSc pathogenesis.

Although many studies have scratched the surface behind the pathogenetic role of disease specific auto-Abs in SSc, there are still a lot to be clarified. Scleroderma mouse models could be used for further experiments regarding the potential pathogenetic involvement of those autoantibodies. So far, SSc specific autoantibodies are mainly used for diagnostic as well as prognostic purposes. Emerging data indicate that SSc autoantibodies could be used in the near future to pinpoint patients with certain pathogenetic characteristics and therefore increased likelihood of responding to a specific treatment. Limited evidence exists regarding their pathogenetic involvement.

#### B Cells as Producers of Novel Autoantibodies With a Distinct Pathogenetic Mechanism

Apart from the above mentioned SSc specific autoantibodies, an expanding amount of evidence indicate that several other autoantibodies may be pathogenetically involved in SSc. In 2006, Baroni et al. reported the presence of agonistic antibodies against platelet derived growth factor receptor (PDGFR) and provided evidence that these antibodies have profibrotic properties and may stimulate fibroblasts to produce collagen (57). However, these findings could not be replicated by subsequent studies, in which no agonistic activity of anti-PDGFR antibodies could be shown in SSc patients (58, 59). Further studies were performed and showed that the same SSc patient could have both stimulatory and non-stimulatory anti-PDGFR autoantibodies (60, 61). Through a series of experiments, the authors showed that stimulatory anti-PDGFR antibodies can be detected only in SSc, whereas non-stimulatory anti-PDGFR antibodies are not disease specific. Further studies are needed before definite conclusions regarding the pathogenetic involvement of anti-PDGFR antibodies in SSc can be drawn.

Autoantibodies against both matrix metalloproteinase-1 (MMP-1) and MMP-3 have been detected in SSc (62–64). Anti-MMP auto-Abs were found to block MMP-1 activity in SSc patients, and this led to decreased collagen degradation and therefore increased deposition in tissues. Furthermore, anti-fibrillin-1 antibodies have been described in SSc patients but are not specific (65, 66). Anti-fibrillin-1 auto-Abs were purified from sera of SSc patients, and it was shown that they had the ability to activate normal fibroblasts *in vitro*. Of note, fibrillin-1 regulates TGF-β secretion therefore anti-fibrillin-1 auto-Abs might contribute to SSc pathogenesis through a TGF-β dependent pathway.

Anti-endothelial cells antibodies (AECA) have long been described in SSc but are not disease specific (67). Two studies from Servettaz et al. and Hill et al. reported that AECAs target CENP-B in lcSSc patients (68, 69). Later on, Arends et al. incubated human umbilical vein endothelial cells with IgGs from AECA (+) SSc patients and controls (70). A significantly higher secretion of ICAM-1, VCAM-1, IL-6, IL-8 and CCL2 was marked in AECA (+) SSc patients compared to AECA (–) patients and controls. In another *in vivo* study, researchers transferred AECA (+) serum from a chicken model of SSc to healthy chicken embryos (71). It was shown that AECAs could bind to microvascular endothelium, leading to increased endothelial cell apoptosis compared to control animals but how these findings could translate to humans remains unknown.

Riemekasten et al. revealed the presence of agonistic auto-Abs against the angiotensin II receptor type 1 (AT1R) and the endothelin receptor type A (ETAR) in SSc patients (72). It was shown that both auto-Abs could bind to their respective receptors on endothelial cells and increase kinase phosphorylation and TGF-β gene expression. Becker et al. revealed inflammatory pulmonary vasculopathy in mice injected with SSc IgGs positive for anti-AT1R and anti-ETAR (73). In the same manner, researchers showed increased IL-8 homologue levels and neutrophil infiltration in BALF from mice injected with SSc IgGs positive for those auto-Abs (74). All these experiments point to a potential role of anti-AT1R and anti-ETAR antibodies in the fibrotic and vascular SSc pathology.

Another antibody of interest in SSc is the one against the muscarinic-3 receptor (M3R). Anti-M3R antibodies have been detected in SSc patients with gastrointestinal (GI) involvement (75). Eaker et al. injected rats with IgG fractions from an anti-M3R (+) SSc patient or controls (76). Intestinal myoelectric activity disturbances were noticed in the rats immunized with the scleroderma IgG. In a similar fashion, Goldblatt et al. assessed the stimulation of mouse colon longitudinal muscle by muscarinic agonists in the presence of IgG fractions derived from patients with SSc or healthy subjects (77). They found that M3R responsible contractions were alleviated by IgGs from SSc patients. These findings were further supported by another research team who proved that M3R activation was blocked in internal anal sphincter smooth muscle cells of rats, when injected with SSc IgGs (78). Results from the studies above, could suggest a role of anti-M3R antibodies in SSc GI dysmotility. The above data suggest that certain autoAbs may participate in pathogenesis and further strengthen the role of B cells in SSc pathophysiology.

#### B Cells as Producers of Profibrotic Cytokines in SSc

B cells can produce several cytokines and growth factors of interest in SSc pathophysiology (79). Dumoitier et al. showed that peripheral B cells from patients with SSc can produce high levels of IL-6 and TGFβ1 (34); these cytokines have well known profibrotic properties (80–82).

In a recent study, Fukasawa et al. analyzed the behavior of B cells in SSc patients and scleroderma mouse model at the single cell level (83). In ATA (+) SSc patients, the affinity of B cells for Topo I determined the cytokine production profile of each B cell. The affinity was defined based on the responsiveness of B cells to Topo I. The affinity was then marked as high or low, based on ATA IgG levels. This categorization subsequently led to the formation of two groups. In the high affinity group, B cells produced significantly higher amounts of the proinflammatory cytokines, IL-6 and IL-23. In contrast, B cells form the low affinity group produced significantly higher amounts of the anti-inflammatory cytokines, IL-10 and IL-35.

#### B Cells Can Interact With Other Cell Types and Promote Fibrosis

Accumulating data indicate that B cells may interact with fibroblasts, dendritic cells as well as macrophages and promote fibrotic responses. In an *in vitro* study, Francois et al. co-cultured B cells and human skin fibroblasts from SSc patients and controls and performed transwell experiments to assess contact- and soluble factor-mediated interactions (84). Both SSc and control fibroblasts produced high levels of collagen when co-cultured with B cells. However, this effect was inhibited with the transwell experiments, pointing to the importance of cell to cell contact in collagen production.

Experimental data indicate that activated B cells may induce dendritic cell maturation *in vitro*; these dendritic cells promote the polarization of Th cells towards the Th2 phenotype and produce the profibrotic cytokines IL-4, IL-5 and IL-13 (85). In a recent study, B cells were extracted from mice with bleomycin induced scleroderma and were co-cultured with macrophages; it was shown that macrophages were skewed towards a profibrotic phenotype. Similarly, B cells from humans with severe dcSSc and associated ILD promoted the expression of CD206 on co-cultured macrophages which indicates a profibrotic response (86).

**Figure 3:**
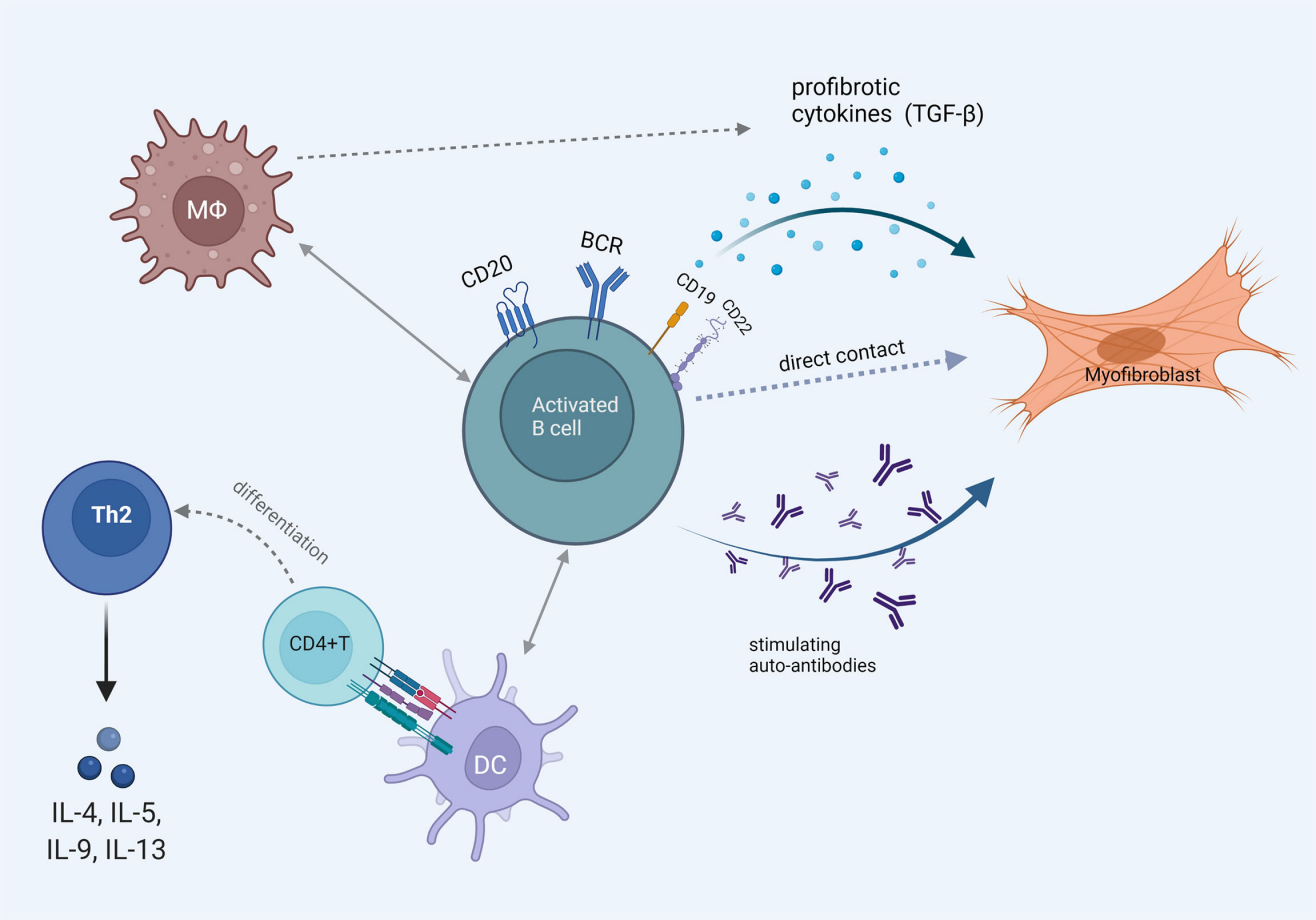
The multiple faces of B cell activation that promotes fibrosis. Pathogenetically involved autoantibody production and B cell direct profibrotic cytokine release. Cell interaction with fibroblasts, macrophages and dendritic cells leading to Th2 mediated profibrotic cytokine release and myofibroblast differentiation.

These data point to the direction that B cells have the ability to interact with many other cells that are of interest in SSc pathophysiology and facilitate the fibrotic process. These are diagrammatically shown in **Figure 3**.

### From Basic Science to Clinical Practice: B Cells as Promising Therapeutic Targets

SSc is a rare systemic disease with various manifestations and challenging therapeutic approach. It is critical to identify molecular and cellular pathways that could be targeted safely and efficiently for our patients (87). Current immunomodulatory therapeutic options include mycophenolate mofetil, cyclophosphamide, tocilizumab and autologous hematopoietic stem cell transplantation (88–93). However, limited efficacy, adverse events, and a still unmet need for better subgroup classification of patients in order to find the best time point and treatment choice to fit each patient, make the continuous quest for new treatments and better therapeutic strategy a necessity.

Targeting B cells can occur either through direct depletion or inhibition of critical cytokines such as the survival factor BAFF and the cytokine IL-6. High levels of IL-6 are produced by B cells in SSc (34), and targeting the receptor of this cytokine with tocilizumab has been studied in SSc. In a randomized placebo-controlled study, tocilizumab was found to preserve lung function in patients with early ILD associated with SSc, even though no significant effect was observed regarding skin fibrosis (90). B cell depletion therapy with rituximab, a chimeric monoclonal antibody against CD20, has been studied in SSc in the past thirteen years from various research teams (94–98). Most studies have shown encouraging results both in skin and lung fibrosis, but their open label design and the small number of participants did not allow definite conclusions to be drawn. Our research group has conducted one of the first trials of RTX in SSc (97). In this proof of principle study, eight patients were randomized to receive 2 cycles of RTX on top of standard treatment and were compared with six patients on standard treatment alone at 1-year timepoint. A significant improvement in lung function was recorded in the RTX group versus the control group. Open label follow up study of the same patients at 2 years, as well as long term data from multiple centers in Greece have shown that continuous treatment with RTX is beneficial and safe for patients with SSc (98, 99). An observational prospective study with a large number of patients treated with RTX from the European Scleroderma Trials and Research (EUSTAR) network, has shown that RTX is a safe choice with a significant effect on skin fibrosis compared with untreated control patients (100). This study did not reveal a significant effect concerning the lung; however, secondary data analyses point to a beneficial effect in lung function when RTX is combined with mycophenolate mofetil. Data from case series also support this combination treatment in progressive ILD disease (101). In an open label, randomized, controlled trial comparing intravenous cyclophosphamide with RTX, the primary outcome of forced vital capacity (FVC) change at 6 months significantly favored the RTX arm. In addition, RTX also improved the modified Rodnan skin score (mRSS) score with a superior safety profile (102).

Several reviews with meta-analyses have been published recently regarding the efficacy of RTX in SSc (103–108). These reviews have differences concerning study inclusion criteria and all suffer from the paucity of high-powered randomized studies. Overall, the results of these meta-analyses indicate that RTX is well tolerated in SSc with promising results both in skin and lung function. B cell depletion treatment is currently an accepted therapeutic choice in resistant disease (109) However, it seems that the time has come to focus on well-designed prospective double-blind studies assessing RTX in SSc. Even though the small double blind randomized controlled trial by Boonstra et al. (110) failed to show a favorable response in lung and skin of patients treated with RTX compared to placebo, the recent DESIRES trial led to the official approval of RTX as treatment for SSc in Japan (111). The DESIRES trial was a multicentric study assessing the use of 375mg/m2 of RTX weekly for four consecutive weeks vs placebo. The study met its primary endpoint, which was the absolute change of the mRSS score at 24 weeks after treatment initiation. Interestingly, FVC also showed a significant improvement, with an initial decline until 12 weeks followed by an increase recorded at 24 weeks’ time-point. On the other hand, FVC had a continuous decline trend in the placebo group. Diffusing capacity of the lung for carbon monoxide (DLCO) stabilization was evident in the RTX arm; however, the comparison with the placebo group did not reach statistical significance. Safety profile was similar between the two groups of patients. A *post-hoc* analysis of this trial recently published, suggests possible baseline biomarkers, which could identify patients with better response to RTX. The authors propose that higher peripheral blood B cell count and mRSS score at baseline could predict a greater improvement in skin sclerosis for RTX treated patients (112). Another placebo controlled randomized study recently published examined a cohort of patients with SSc-associated pulmonary arterial hypertension (PAH) (113). Patients with significant ILD were excluded and primary endpoint was the change in the 6 min-walk distance (6MWD) from baseline at 24 weeks. 57 patients on standard PAH therapy were randomized to receive either 2 infusions of 1gr RTX at baseline or placebo. This phase 2 study failed to reach its endpoints, however, *post hoc* analysis of the available data revealed positive results for RTX and the 6MWD change at the 48 weeks’ time point. In addition, applying a biomarker including low levels of IL-17, IL-12 and rheumatoid factor predicted response to RTX, suggesting that this “negative trial” could be indeed promising for RTX in a subset of patients with SSc-PAH (114).

## Discussion

A significant amount of experimental evidence points to the direction that B cell homeostasis in SSc is dysregulated (115–117). B cells in SSc display not only phenotypic changes but are characterized by changes in intracellular signaling; moreover, they infiltrate affected tissues especially at the early stage of disease. B cells can also produce autoantibodies or cytokines that may promote the fibrotic process. Based on these data, B cell targeted therapies have already been tested with promising results. Until now, we have limited evidence on how B cell depletion may exert its potential clinical efficacy in SSc. One may hypothesize that RTX may diminish the production of pathogenetic autoAbs but this is not supported by data showing that clinical improvement with RTX appears prior to any effect on autoAbs (111). Experimental evidence indicates that B cell depletion in patients with SSc associates with a reduction in PDGFR expression and activation in skin fibroblasts (118). Moreover, SSc patients responding to RTX treatment exhibit an upregulation of Dickkopf-1 (Dkk-1), a key inhibitor of the profibrotic Wnt pathway; of note, Dkk-1 is strikingly absent from SSc skin (119, 120). These data underlie the complex interaction of B cells with multiple other cells as well as molecular pathways. The most critical question is not how RTX may mediate its potential beneficial effects but whether we have the tools to predict which patients are more likely to respond to B cell targeted therapies. From the first clinical trials of RTX in SSc it was evident that patients who had higher skin B cell counts at baseline that were depleted following RTX treatment were the ones with the best clinical response (97). However, skin biopsy is not easily performed in patients with SSc in everyday clinical practice. Therefore, data coming from the recent DESIRES trial indicating that circulating CD19+ cell count could serve as reliable predictor of response to RTX, are of major clinical significance. RTX may be an attractive therapeutic approach when there is no response to standard treatment with mycophenolate mofetil, especially in patients with early disease (121) and high CD19+ cell counts.

## Author Contributions

KM performed the literature search and drafted the manuscript jointly with DD. GI assisted in literature search and manuscript drafting. LS assisted in literature search and manuscript drafting. DD conceived the idea of the review, assisted in literature search, analyzed data, coordinated the project and drafted the manuscript jointly with KM.

## Funding

Publication fees were funded by the University of Patras Research Committee.

## Conflict of Interest

The authors declare that the research was conducted in the absence of any commercial or financial relationships that could be construed as a potential conflict of interest.

## Publisher’s Note

All claims expressed in this article are solely those of the authors and do not necessarily represent those of their affiliated organizations, or those of the publisher, the editors and the reviewers. Any product that may be evaluated in this article, or claim that may be made by its manufacturer, is not guaranteed or endorsed by the publisher.
